# Utility of remnant liver volume for predicting posthepatectomy liver failure after hepatectomy with extrahepatic bile duct resection

**DOI:** 10.1093/bjsopen/zraa049

**Published:** 2021-02-15

**Authors:** R Yamamoto, T Sugiura, Y Okamura, T Ito, Y Yamamoto, R Ashida, K Ohgi, S Otsuka, K Uesaka

**Affiliations:** Division of Hepato-Biliary-Pancreatic Surgery, Shizuoka Cancer Centre, Shizuoka, Japan

## Abstract

**Background:**

Hepatectomy with extrahepatic bile duct resection is associated with a high risk of posthepatectomy liver failure (PHLF). However, the utility of the remnant liver volume (RLV) in cholangiocarcinoma has not been studied intensively.

**Methods:**

Patients who underwent major hepatectomy with extrahepatic bile duct resection between 2002 and 2018 were reviewed. The RLV was divided by body surface area (BSA) to normalize individual physical differences. Risk factors for clinically relevant PHLF were evaluated with special reference to the RLV/BSA.

**Results:**

A total of 289 patients were included. The optimal cut-off value for RLV/BSA was determined to be 300 ml/m^2^. Thirty-two patients (11.1 per cent) developed PHLF. PHLF was more frequent in patients with an RLV/BSA below 300 ml/m^2^ than in those with a value of 300 ml/m^2^ or greater: 19 of 87 (22 per cent) *versus* 13 of 202 (6.4 per cent) (*P *<* *0.001). In multivariable analysis, RLV/BSA below 300 ml/m^2^ (*P *=* *0.013), future liver remnant plasma clearance rate of indocyanine green less than 0.075 (*P *=* *0.031), and serum albumin level below 3.5 g/dl (*P *=* *0.015) were identified as independent risk factors for PHLF. Based on these risk factors, patients were classified into three subgroups with low (no factors), moderate (1–2 factors), and high (3 factors) risk of PHLF, with PHLF rates of 1.8, 14.8 and 63 per cent respectively (*P *<* *0.001).

**Conclusion:**

An RLV/BSA of 300 ml/m^2^ is a simple predictor of PHLF in patients undergoing hepatectomy with extrahepatic bile duct resection.

## Teaser

The present study showed the importance of considering remnant liver volume (RLV)/body surface area (BSA) in addition to indocyanine green test for hepatectomy with extrahepatic bile duct resection. An RLV/BSA of 300 ml/m^2^ is a simple and useful cut-off value for predicting posthepatectomy liver failure in patients undergoing major hepatectomy for cholangiocarcinoma.

## Resumen

**Antecedentes:**

La hepatectomía con resección de la vía biliar extrahepática se relaciona con un riesgo elevado de insuficiencia hepática post-hepatectomía (*posthepatectomy liver failure*, PHLF). Sin embargo, la utilidad del volumen del remanente hepático (*remnant liver volumen*, RLV) no se ha analizado en profundidad en el colangiocarcinoma.

**Métodos:**

Se revisaron los pacientes sometidos a una hepatectomía mayor con resección de la vía biliar extrahepática entre 2002 y 2018. Para normalizar las diferencias físicas individuales, el RLV se dividió por la superficie corporal (*body surface area*, BSA). Se estudiaron los factores de riesgo de PHLF clínicamente relevante con énfasis en la relación RLV/BSA.

**Resultados:**

Se incluyeron 289 pacientes. El valor de corte óptimo del RLV/BSA fue de 300 mL/m^2^. Treinta y dos pacientes (11,1%) desarrollaron PHLF. La PHLF fue más frecuente en pacientes con un RLV/BSA < 300 mL/m^2^ (19 de 87 (21,8%)) que en aquellos con un RLV/BSA ≥ 300 mL/m^2^ (13 de 202 (6,4%), *P* < 0,001). En un análisis multivariante, se identificaron como factores de riesgo independientes de PHLF: un RLV/BSA <300 mL/m^2^ (*P* = 0,013), una tasa de aclaramiento plasmático de verde de indocianina del remanente hepático < 0,075 (*P* = 0,031) y la albúmina sérica < 3,5 g/dL (*P* = 0,015). De acuerdo con estos factores de riesgo, los pacientes se clasificaron en 3 subgrupos de riesgo: bajo (ningún factor), moderado (1-2 factores) y alto (3 factores) con tasas de PHLF del 1,8%, 14,8% y 62,5%, respectivamente (*P* < 0,001).

**Conclusión:**

Un RLV/BSA de 300 mL/m^2^ es un predictor sencillo de PHLF en pacientes sometidos a hepatectomía con resección de la vía biliar extrahepática.

## Introduction

Posthepatectomy liver failure (PHLF) is one of the most serious complications after major hepatectomy and closely related to perioperative mortality^1^. In particular, hepatectomy with extrahepatic bile duct resection is associated with a high risk of PHLF compared with hepatectomy without bile duct resection^2^. Although high-volume centres in Japan have recently reduced mortality rates to below 5 per cent with advances in surgical technique and perioperative management methods, PHLF remains a life-threating complication after major hepatectomy with extrahepatic bile duct resection^3^^,^^4^.

PHLF is closely associated with liver functional reserve, which should be evaluated by both liver function and future remnant liver volume (RLV)^5^. Clinical practice guidelines for the management of biliary tract cancers (3rd edition)^6^ published by the Japanese Society of Hepato-Biliary-Pancreatic Surgery recommend using the future liver remnant plasma clearance rate of indocyanine green (ICGK-F), which is calculated as plasma clearance rate of indocyanine green (ICGK) multiplied by the remnant liver proportion measured by CT volumetry, to evaluate the functional reserve of the remnant liver. Although the cut-off value for ICGK-F has been set at 0.05 to reduce the risk of PHLF and death after liver resection of cholangiocarcinoma^7^^,^^8^, ICGK-F alone was unable to predict PHLF sufficiently^9^. Therefore, complementary factors should be considered in addition to ICGK-F in order to predict PHLF more precisely and to decide the surgical indication more appropriately. The RLV is intuitive and easily determined by CT volumetry^10^. Several studies^10–12^ reported that the normalized RLV was a useful indicator for prediction of PHLF following hepatectomy. However, the importance of normalized RLV in biliary tract cancer has not been studied intensively, except in one recent report^13^.

The purpose of this study was to assess the value of parenchymal RLV in addition to ICGK-F in predicting PHLF after hepatectomy with extrahepatic bile duct resection for cholangiocarcinoma. There are several methods for normalization of parenchymal RLV, principally with reference to body surface area (BSA)^10^ or bodyweight^13^. It is known that BSA correlates closely with liver volume^14^, and that BSA has long been used to calculate the normalized liver volume for liver surgery in the clinical setting^10^^,^^11^. Moreover, Hashimoto and colleagues^15^ reported that BSA was the most powerful indicator for estimating liver volume in Japanese individuals. To this end, RLV/BSA was used to allow for individual liver volume differences in the present study.

## Methods

This was a retrospective review of a prospectively maintained cholangiocarcinoma database. Consecutive patients who underwent major hepatectomy (resection of 3 or more Couinaud’s segments)^16^ with extrahepatic bile duct resection for cholangiocarcinoma between September 2002 and December 2018 in Shizuoka Cancer Centre were analysed. This study was approved by the institutional ethics committee (approval number J2019-143-2019-1-3) and reported in accordance with the STROBE statement^17^.

### Preoperative management

Preoperative biliary drainage was undertaken routinely in patients with jaundice via an endoscopic or percutaneous transhepatic approach. Biliary drainage was usually performed for the unilateral biliary tree of the future liver remnant to enhance functional reserve, whereas bilateral drainage was considered when uncontrolled cholangitis developed or the total serum bilirubin concentration was more than 2.0 mg/dl even after unilateral drainage^18^. The indocyanine green (ICG) test was used when the total serum bilirubin level dropped below 2.0 mg/dl^18^.

CT volumetry was done by hand-tracing axial CT or using a SYNAPSE VINCENT image analysis system (Fujifilm, Tokyo, Japan), with liver volume calculated as the volume of hepatic parenchyma minus the volume of the tumour and major vessels. In this hospital, an ICGK-F value of 0.05 was adopted as the cut-off value for deciding on liver resection^19^. Portal vein embolization (PVE)^18^ was performed routinely when the ICGK-F value was below 0.05. However, even if it was 0.05 or higher, PVE was undertaken when the remnant liver proportion was less than 40 per cent, and in patients undergoing trisectionectomy or right hepatectomy. CT volumetry and ICG tests were carried out 2–3 weeks after PVE to re-evaluate the RLV and ICGK-F^18^.

Hepatectomy was performed when the criterion of ICGK-F at least 0.05 was met. If this criterion was not met, the operation was postponed; CT volumetry and ICG tests were then repeated every 2–4 weeks until the ICGK-F value exceeded 0.05. Patients whose ICGK-F value remained below 0.05 after a long period did not usually undergo hepatectomy, although in some exceptional cases hepatectomy was undertaken when patient requested surgical resection with appropriate informed consent. The Pringle manoeuvre was routinely performed during hepatectomy.

BSA was used to normalize individual liver volume differences. BSA was calculated according to the Du Bois formula^20^: BSA (m^2^)=weight (kg)0^.425^×height (cm)0^.725^×0.007184. RLV/BSA had not been used to decide on liver resection in medical practice before now, and was calculated for the present study. In contrast, the standardized functional liver remnant (sFLR)^21^^,^^22^, defined as the remnant liver volume as a proportion of estimated total liver volume (1267.28 × BSA – 794.41), was calculated to compare the receiver operating characteristic (ROC) curve with that for RLV/BSA.

### Definition of postoperative complications

PHLF was defined according to the criteria of the International Study Group of Liver Surgery (ISGLS)^1^. The present study concerned clinically relevant PHLF, which included only grades B and C. Grade A PHLF was included in the no-PHLF group because it is clinically unimportant. Postoperative complications were evaluated according to the Dindo–Clavien classification^23^. Surgical-site infection, ascites, and pleural effusion with a Clavien–Dindo grade of II or more were considered. Bile leakage was evaluated according to the ISGLS criteria^1^.

### Statistical analysis

Continuous variables are presented as median (range), with analysis using the Mann–Whitney *U* test. Categorical variables were analysed by Fisher’s exact test. Correlation was evaluated by Spearman rank correlation coefficient. Cut-off values for continuous variables were established from ROC curve analyses. Logistic regression was used for multivariable analyses to identify risk factors for PHLF. Two-sided *P* < 0.050 was considered statistically significant. Statistical analyses were performed using SPSS^®^ version 19⋅0 (IBM, Armonk, New York, USA).

## RESULTS

A total of 306 patients underwent major hepatectomy with extrahepatic bile duct resection for cholangiocarcinoma. Eight patients who did not undergo CT volumetry or ICG test were excluded. A further nine patients who experienced troublesome complications related to the operative procedure (portal vein occlusion in 4, arterial trauma in 3, fatal duodenal leakage in 1, and intraoperative septic shock in 1) were also excluded in order to purely evaluate predictive factors for PHLF. After excluding these 17 patients, 289 were included in the retrospective analysis.

**Fig. 1:**
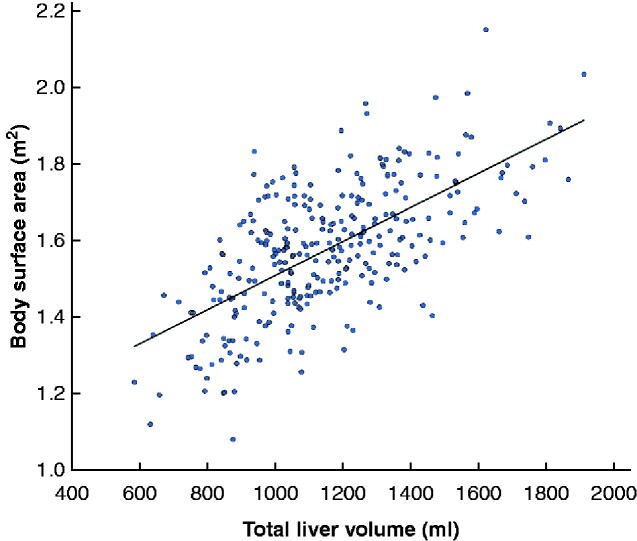
Correlation between body surface area and total liver volume Correlation coefficient 0.620, *P *<* *0.001.

Some 146 of the 289 patients (50.5 per cent) underwent preoperative PVE to increase the RLV. In such patients, the RLV significantly increased from median 369 (range 170–977) ml before PVE to 465 (250–1120) ml after embolization (*P *<* *0.001). On CT volumetry just before hepatectomy, the median RLV among all patients was 549 (250–1416) ml, and the total liver volume was 1166 (647–2040) ml. The median BSA was 1.58 (1.08–2.14) m^2^; BSA correlated significantly with total liver volume (correlation coefficient 0.620, *P *<* *0.001) (*Fig. 1*).

Patient characteristics according to PHLF are described in *Table 1*. Clinically relevant PHLF developed in 32 patients (11.1 per cent): grade B in 25 and grade C in seven patients. The no-PHLF group consisted of 257 patients, including 18 with grade A PHLF. Serum albumin values just before hepatectomy were significantly lower in the PHLF group than in the no-PHLF group. The proportion of patients with chronic hepatitis did not differ markedly by presence of PHLF, and the disease in all patients was classified as Child–Pugh A. RLV and ICGK-F were significantly smaller in the PHLF group.

**Table 1:** Patient characteristics according to presence of posthepatectomy liver failure

	No PHLF (*n* = 257)	**PHLF** **(*n* = 32)**	***P*** ^‡^
**Preoperative characteristics**			
Age (years)^*^	70 (32–87)	71 (52–87)	0.043^§^
Sex ratio (M : F)	178 : 79	24 : 8	0.683
BSA (m^2^)^*^	1.58 (1.08–2.03)	1.58 (1.21–2.14)	0.916^§^
Albumin (g/dl)^*^	4.0 (2.3–5.2)	3.7 (2.8–4.6)	0.022^§^
Total bilirubin (mg/dl)^*^	0.8 (0.3–2.2)	0.8 (0.4–1.8)	0.074^§^
Platelet count (×10^3^/μl)^*^	24 (10–78)	23 (14–41)	0.380^§^
Diabetes mellitus	37 (14.4)	7 (22)	0.295
Chronic hepatitis	8 (3.1)	2 (6)	0.306
Preoperative cholangitis	54 (21.0)	10 (31)	0.184
Preoperative biliary drainage	175 (68.1)	24 (75)	0.545
Portal vein embolization	125 (48.6)	21 (66)	0.091
ICGK^*^	0.151 (0.095–0.321)	0.151 (0.137–0.272)	0.904^§^
ICGK-F^*^	0.078 (0.032–0.234)	0.064 (0.038–0.098)	< 0.001^§^
RLV (ml) *	580 (259–1416)	429 (250–902)	0.001^§^
RLV/BSA (ml/m^2^)^*^	359 (163–966)	284 (167–537)	< 0.001^§^
Remnant liver proportion (%)^*^	49 (24–95)	40 (28–72)	< 0.001^§^
sFLR (%)^*^	47 (21–141)	39 (22–71)	< 0.001^§^
**Intraoperative outcomes**			
Type of hepatectomy			0.011
Right hepatectomy	91 (35.4)	14 (44)	
Left hepatectomy	100 (38.9)	4 (13)	
Right trisectionectomy	19 (7.4)	6 (19)	
Left trisectionectomy	42 (16.3)	7 (22)	
Central bisectionectomy	5 (1.9)	1 (3)	
Duration of Pringle manoeuvre (min)^*^	49 (15–151)	34 (20–109)	0.628^§^
Vascular resection	90 (35.0)	11 (34)	1.000
Pancreatoduodenectomy	72 (28.0)	8 (25)	0.836
Duration of operation (min)^*^	485 (195–973)	578 (344–1171)	0.622^§^
Blood loss (g)^*^	1420 (354–12671)	1495 (585–7788)	0.320§
Blood transfusion	60 (23.3)	12 (38)	0.087
**Postoperative outcomes**			
Morbidity (grade ≥ III)^†^	119 (46.3)	25 (78)	< 0.001
Surgical-site infection (grade ≥ II)^†^	50 (19.5)	16 (50)	< 0.001
Ascites (grade ≥ II)^†^	34 (13.2)	15 (47)	< 0.001
Pleural effusion (grade ≥ II)^†^	4 (1.6)	10 (31)	< 0.001
Bile leakage (ISGLS)	40 (15.6)	12 (38)	0.006
Reoperation	2 (0.8)	4 (13)	0.002
Death	1 (0.4)	3 (9)	0.005

Values in parentheses are percentages unless otherwise indicated;

* values are median (range).

† Grade according to Dindo–Clavien classification. PHLF, posthepatectomy liver failure; BSA, body surface area; ICGK, plasma clearance rate of indocyanine green (ICG); ICGK-F, future liver remnant plasma clearance rate of ICG; RLV, remnant liver volume; sFLR, standardized functional liver remnant; ISGLS, International Study Group of Liver Surgery.

‡ Fisher’s exact test, except

§ Mann–Whitney *U* test.

Right trisectionectomy was performed more frequently, and left hepatectomy less frequently, in the PHLF group. There were no significant differences in duration of operation, operative blood, loss, and blood transfusion between the groups. Postoperative complications and reoperation were more common in the PHLF group.

**Fig. 2:**
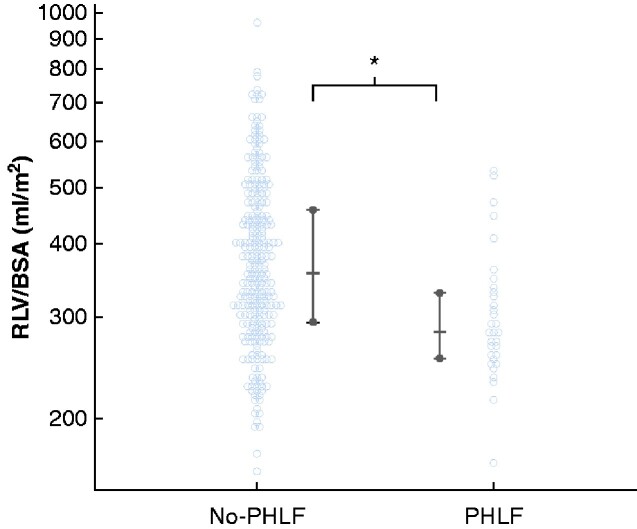
Distribution of patients according to posthepatectomy liver failure Values are shown for individual patients, and summarized as median with interquirtile range. RLV, remnant liver volume; BSA, body surface area; PHLF, posthepatectomy liver failure. *P <* *0.001 (Mann–Whitney *U* test).

**Fig. 3:**
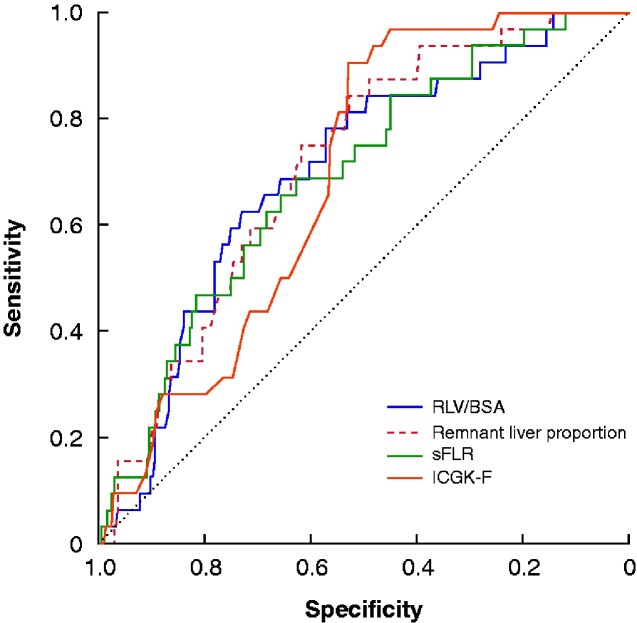
Receiver operating characteristic (ROC) curves for predicting posthepatectomy liver failure The cut-off value for remnant liver volume (RLV)/body surface area (BSA) was 300 ml/m^2^ (area under the curve (AUC) 0.699, sensitivity 62.5 per cent, specificity 73.2 per cent). The cut-off value for remnant liver proportion was 47.6 per cent (AUC 0.713, sensitivity 52.9 per cent, specificity 84.4 per cent). The cut-off value for standardized functional liver remnant (sFLR) was 42.8 per cent (AUC 0.690, sensitivity 62.6 per cent, specificity 68.8 per cent). The cut-off value for future liver remnant plasma clearance rate of indocyanine green (ICGK-F) was 0.075 (AUC 0.689, sensitivity 52.9 per cent, specificity 90.6 per cent).

RLV/BSA was significantly smaller in the PHLF group: median 284 (167–537) ml/m^2^*versus* 359 (163–966) ml/m^2^ in the no-PHLF group (*Fig. 2*). In ROC curve analysis, the optimal cut-off value of RLV/BSA for predicting PHLF was 300 ml/m^2^ (area under the curve (AUC) 0.699, sensitivity 62.5 per cent, specificity 73.2 per cent) (*Fig. 3*). The RLV/BSA value was below 300 ml/m^2^ in 87 patients (30.1 per cent), and 300 ml/m^2^ or higher in 202 (79.9 per cent). ROC curves for remnant liver proportion (AUC 0.713; *P *=* *0.658), sFLR (AUC 0.690; *P *=* *0.599) and ICGK-F (AUC, 0.689; *P *=* *0.827) were not markedly different from that for RLV/BSA.

Based on the cut-off for RLV/BSA, PHLF developed more frequently in patients with an RLV/BSA of 300 ml/m^2^ than in those with a higher value: 19 of 87 (22 per cent) *versus* 13 of 202 (6.4 per cent) (*P *<* *0.001). During the study interval, 32 patients (11.1 per cent) with an ICGK-F value below 0.05 underwent major hepatectomy under appropriate informed consent. Of these, 6 of 24 patients with an RLV/BSA below 300 ml/m^2^ experienced PHLF, compared with none of eight with an RLV/BSA of 300 ml/m^2^ or higher. Four of 289 patients (1.4 per cent) died from postoperative morbidity: PHLF in three patients and aspiration pneumonia in one. The RLV/BSA values for the three patients with PHLF were 167, 249, and 314 ml/m^2^ respectively.

### Preoperative and intraoperative risk factors for posthepatectomy liver failure

**Fig. 4:**
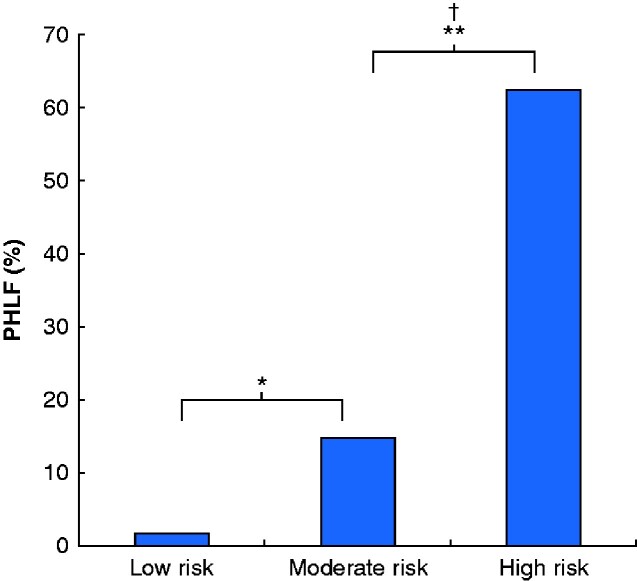
Rate of posthepatectomy liver failure according to risk classification based on independent risk factors in multivariable analysis Cut-off values for independent risk factors were: remnant liver volume/body surface area less than 300 ml/m^2^, future liver remnant plasma clearance rate of indocyanine green less than 0.075, and serum albumin concentration less than 3.5 g/dl. Low risk, no factors; moderate risk, 1–2 factors; high risk, 3 factors. *P =* *0.001, †*P *=* *0.012 (adjusted Fisher’s exact test using Bonferroni method).

ROC curve analyses identified the following cut-off values: serum albumin, 3.5 g/dl; ICGK-F, 0.075; age, 75 years; duration of operation, 650 min; and blood loss, 1500 g. These values were entered into univariable and multivariable analyses. The multivariable analysis identified serum albumin value below 3.5 g/dl (odds ratio (OR) 3.22), ICGK-F less than 0.075 (OR 3.07) and RLV/BSA less than 300 ml/m^2^ (OR 2.94) as independent risk factors for PHLF (*Table 2*). Patients were classified into three subgroups according to these risk factors with similar ORs: low risk (no factor, 112 patients), moderate risk (1–2 factors, 169 patients), and high risk (3 factors, 8 patients). PHLF was significantly associated with this risk classification; PHLF developed in two low-risk patients (1.8 per cent), 25 moderate-risk patients (14.8 per cent), and five high-risk patients (63 per cent). (*P *<* *0.001) (*Fig. 4*).

**Table 2:** Results of logistic regression analysis to identify risk factors for posthepatectomy liver failure

	No. of patients	Univariable analysis		Multivariable analysis	
		Odds ratio	*P*	Odds ratio	*P*
**Age (years)**			0.087		
< 75	209	1.00 (reference)			
≥ 75	80	1.94 (0.91, 4.14)			
**Albumin (g/dl)**			0.030		0.013
< 3.5	43	2.57 (1.10, 6.01)		3.22 (1.28, 8.11)	
≥ 3.5	246	1.00 (reference)		1.00 (reference)	
**Portal vein embolization**			0.074		
No	143	1.00 (reference)			
Yes	146	2.02 (0.93, 4.35)			
**ICGK-F**			< 0.001		0.031
< 0.075	146	4.95 (1.97, 12.4)		3.07 (1.11, 8.52)	
≥ 0.075	143	1.00 (reference)		1.00 (reference)	
**RLV/BSA (ml/m^2^)**					0.015
< 300	87	4.06 (1.90, 8.67)	< 0.001	2.94 (1.23, 7.01)	
≥ 300	202	1.00 (reference)		1.00 (reference)	
**Duration of operation (min)**			0.809		
< 650	204	1.00 (reference)			
≥ 650	85	1.10 (0.50, 2.44)			
**Blood loss (g)**			0.546		
< 1500	159	1.00 (reference)			
≥ 1500	130	1.25 (0.60, 2.62)			
**Blood transfusion**			0.085		
No	217	1.00 (reference)			
Yes	72	1.97 (0.91, 4.26)			

Values in parentheses are 95 per cent confidence intervals. ICGK-F, future liver remnant plasma clearance rate of indocyanine green; RLV, remnant liver volume; BSA, body surface area.

## Discussion

Surgical resection for cholangiocarcinoma requires massive hepatectomy with extrahepatic bile duct resection, which is associated with a high risk of PHLF^2^^,^^24^. To reduce the risk of liver failure after major hepatectomy, the future liver remnant has usually been evaluated based on the proportion of remnant liver to total liver volume^5^^,^^18^. When the remnant liver proportion is less than 30–40 per cent, PVE is performed at high-volume centres of hepatobiliary surgery^18^^,^^25^. The remnant liver proportion and ICG test have been used commonly in Eastern countries^18^^,^^26^, whereas the sFLR has been used in Western countries^21^^,^^22^. However, the normalized RLV has not been considered for patients undergoing hepatectomy for biliary tract cancer^13^. The concept of the RLV/BSA is intrinsically different from that of the remnant liver proportion, as the RLV/BSA is the normalized parenchymal volume of the remnant liver rather than a proportion. The present study showed that the AUC for the RLV/BSA was comparable to that for the remnant liver proportion and sFLR, and underscored the importance of considering RLV/BSA in addition to ICGK-F (ICGK × remnant liver proportion) for hepatectomy with extrahepatic bile duct resection. An RLV/BSA of 300 ml/m^2^ is a simple and useful cut-off value for prediction of PHLF in patients undergoing major hepatectomy with extrahepatic bile duct resection.

Two decades ago, Shirabe and colleagues^10^ studied the association between RLV and PHLF in 80 patients who underwent major hepatectomy for hepatocellular carcinoma, and reported that the cut-off value of RLV/BSA for predicting PHLF was 250 ml/m^2^. Subsequently, Hirashita and co-workers^11^ considered the RLV in 80 patients having right-sided hepatectomy, and also reported that an RLV/BSA value of less than 250 ml/m^2^ was the optimal cut-off. The cut-off value of 250 ml/m^2^ was lower than that of 300 ml/m^2^ in the present study owing to differences in the definition of PHLF and the extent of surgical stress. Hepatectomy with extrahepatic bile duct resection is associated with a high risk of PHLF compared with hepatectomy without bile duct resection^2^. Lee *et al.*^13^ showed that RLV/weight predicted clinically relevant PHLF after hepatectomy for perihilar cholangiocarcinoma, with a cut-off value of 0.5 per cent. However, in the present cohort, an RLV/weight was below 0.5 per cent in only three patients, which might be due to the small physical size of Japanese people^15^^,^^22^. This study employed the RLV/BSA because BSA was reported to be the best indicator for estimating liver volume in Japan^15^. PHLF was more common in patients with an RLV/BSA below 300 ml/m^2^ than in those with a higher value (22 *versus* 6.4 per cent; *P *<* *0.001), and multivariable analysis identified RLV/BSA below 300 ml/m^2^ as an independent risk factor for PHLF. To prevent PHLF, patients with an RLV/BSA below 300 ml/m^2^ should undergo preoperative treatment to increase the RLV/BSA to 300 ml/m^2^, such as PVE^8^ or radiological simultaneous portal vein and hepatic vein embolization^27^.

Future liver functional reserve should be evaluated based not only on RLV but also on liver function. ICGK-F, which combines function and remnant liver proportion, was used to predict PHLF and mortality after hepatectomy for biliary tract cancer in Japan^6–8^. The cut-off value for ICGK-F of 0.075 determined in the present study differed from that in the authors’ clinical setting (ICGK-F 0.05). The former value was determined with the aim of predicting PHLF. This value is similar to that reported by Yokoyama and colleagues^9^, who targeted PHLF. The latter value was determined with the aim of predicting postoperative mortality^7^. The difference in the target might explain the difference between cut-off values.

In the present study, 146 of 289 patients (50.5 per cent) had an ICGK-F below 0.075, of whom 26 (17.8 per cent) developed PHLF. If the cut-off value of ICGK-F had been set at 0.075, half of the patients would have lost the opportunity to receive curative resection. Among these patients, PHLF was more frequently observed in high-risk patients (5 of 8) than in moderate-risk patients (21 of 138; *P *=* *0.005). Therefore, the indication for hepatectomy should be considered based on a risk classification that includes ICGK-F less than 0.075, RLV/BSA below 300 ml/m^2^, and serum albumin value less than 3.5 g/dl. Furthermore, this risk classification can allow expansion of the surgical indication to hepatectomy with extrahepatic bile duct resection. Patients conventionally considered at high risk of developing PHLF based on an insufficient ICGK-F underwent hepatectomy safely when the RLV/BSA was 300 ml/m^2^ or higher. It therefore seems extremely important to consider the ICGK-F, RLV/BSA, and serum albumin value concurrently in preoperative evaluation of the risk of PHLF.

The present study was limited by its retrospective nature, small sample size, and single-centre setting. The value of the RLV/BSA was based on the fact that the BSA was the most powerful indicator for estimating the liver volume in Japanese individuals. Thus, the applicability of the findings of the present study is limited to Japanese patients, and the findings are not necessarily generalizable to Western patients.
